# COVID-19 Communication and Media: The First Pandemic of the Digital Age

**DOI:** 10.1177/00027642231155381

**Published:** 2023-03-01

**Authors:** Jeremy Schulz, Laura Robinson, Massimo Ragnedda, Cara Chiaraluce, Oliver Kleinmann

**Affiliations:** 1 Institute for the Study of Societal Issues, UC Berkeley, Berkeley, CA, USA; 2 Department of Sociology, Santa Clara University, Santa Clara, CA, USA; 3 Media and Communications, Northumbria University, Newcastle upon Tyne, UK; 4 The University of Chicago, Chicago, IL, USA

**Keywords:** Zoom, YouTube, Twitter, COVID-19, pandemic

## Abstract

This issue of *American Behavioral Scientist* marshals case studies of online media platforms such as Zoom, YouTube, and Twitter and digital hardware systems such as virtual reality technology to assess the often unexpected interactions between the pandemic and digital technologies. The issue leads with a case study of Zoom to examine the laregely successful efforts which Zoom made in the wake of the pandemic to resolve unanticipated privacy and security problems afflicting the suddenly ubiquitous and indispensable platform. Subsequently, the issue charts the growing tensions between competing proprietary and open-source institutional logics during the pandemic. In the next section, articles consider the spread of covid-related information on YouTube and news outlets to take a comparative angle of vision both internationally and in terms of the dynamics of media production and reception in different cultural and societal environments. Variation is also key to the articles in the last section where research focuses on persistent digital usage gaps. Here the articles touch on the socioeconomic factors driving differentiated knowledge about the pandemic, as well as the relatively low uptake of digital technologies among older adults in housing facilities. Finally, we also learn about the effect of the social isolation and anxieties of the pandemic on the uptake of a new form of digital hardware, virtual reality equipment. Finally, the issue closes with an eye to visualization tools needed for the future to close this discussion of the digitization of the 100-year crisis occasioned by the COVID-19 pandemic. These contributions take the measure of how the pandemic intersected with digitized communications and media in varied and, at times, unequal ways, as well as lessons applicable to future crises.

This issue of *American Behavioral Scientist* assesses the landscapes of digital media as it was transformed by the first pandemic of the digital age. The case studies in the issue probe some of the most significant digital tools and social media used during this 100-year event including Zoom, YouTube, and Twitter, as well as the examination of the role of digital inequalities and divides in responses to the pandemis, and the potential for new tools including VR and visualizations. Together, the articles facilitate the assessment of some of the important lessons of the pandemic as they apply to the development and mobilization of digital communications and media in the face of future crises.

The issue leads with an examination of Zoom, which is entitled “Why Zoom is not Doomed.” This first article takes a case study approach to examine the unusual response of the videoconferencing company Zoom to the abrupt and unexpected privacy and security problems afflicting the suddenly ubiquitous and indispensable platform. Using transcript materials from Zoom’s official communications with external constituencies, the study identifies the organizational narratives adopted and promoted by Zoom’s leadership in the wake of the crisis. From these communications it is apparent that the firm made an effort to convince external constituencies that it was adapting its business strategy to meet the needs of users concerned about security and privacy. In this way, it took advantage of the crisis as an opportunity for growth and reinvention. This tactic largely helped Zoom to strengthen its organizational mission and culture in spite of the seemingly dire situation. In addition, in appearing to successfully cope with an urgent crisis, the company rebuilt its brand as a reliable, trustworthy platform and its position as an industry standard-bearer.

Whereas the previous articles deal with the responses of specific organizations and sets of organizations, the second article takes a broader view, scrutinizing the organizational field of the internet in its entirety. This contribution, entitled “Navigating Pandemic Crises: Encountering the Digital Commons,” charts the growing tensions between competing proprietary and open-source institutional logics during the pandemic. As the article contends, the internet has always hosted multiple countervailing institutional logics, the logic of the commercialized internet run by capitalist entities, the logic of the government-run internet often used to keep track of citizens, and the “commons” logic best exemplified by the open source software movement. During the pandemic, the tension between these competing logics only grew stronger, as an unprecedented number of activities moved to online platforms and domains and the internet became more indispensable for more people. At the same time, as for-profit firms perfected their techniques of value extraction based on user surveillance, publicly minded entities offered an abundance of content via open access websites and platforms. Advocates of open science and education in particular disseminated large volumes of health information and developed educational tools for open access to individuals around the world. In this way the pandemic created the space for the digital commons logic to flourish, in the online sphere even as the proprietary and capitalist logic often took center stage in the public eye.

In the next section, the research explores the dissemination of information during the early pandemic. The first article expands on the theme of communication during the pandemic by investigating the genres and emotional characteristics of the types of messaging employed in order to motivate behavior during the pandemic across several of the most important countries across the globe. The article “A cross-national Study of Fear Appeal Messages in YouTube Trending Videos about COVID-19” applies the Extended Parallel Process Model to explore the prevalence and psychological content of over 2000 trending videos posted on YouTube across six countries from January to May 2020. The analysis reveals important cross-national differences: while pandemic-themed videos gained early attention in Taiwan, they encountered a prolonged delay in the United States and Brazil. Specifically, COVID-19 videos featured the least prominently in Brazil’s trending list, with most of those spotlighted videos coming from the entertainment category. Where the thematic and psychological content is concerned, the results from the automated content analysis further suggested that fear content vastly overshadowed efficacy content, particularly in countries such as Russia and Brazil, where the collective response to the pandemic was particularly inadequate. These results suggest that governments and other collective actors should strive to create public health messaging that contains more efficacy-inducing content and less fear-inducing content in the future.

Another study attends to multiple sources of variation in the extent to which U.S. adults possess factually correct knowledge of issues around the pandemic. In “Paying Attention to the Pandemic: Knowledge of COVID-19 Facts by News Source and Demographics,” several sources of individual-level variation are subjected to scrutiny, namely sociodemographic and socioeconomic differences across individuals and the relative consumption of different forms of news content relevant to the topics under study. Based on an analysis of nationally representative data drawn from the Pew Foundation, the article reveals that knowledge of facts relating to the pandemic varies according to both of these factors. Logistic models pinpoint differences in the likelihood of correct knowledge among respondents, depending on respondent attributes such as race and income, but also the types of news content which predominate in their news consumption mix. Correlations with race/ethnicity, education, and income indicate how the U.S. current information ecosystem caters to higher SES information consumers, suggesting that respondents who rely more heavily on informal news sources and local news sources tend to lack correct knowledge when compared to those who depend more on national news outlets and government sources.

While the first two sections survey the imperfect ways digital technologies were marshaled to contend with issues raised by the pandemic. In the last section we turn to potential new avenues and solutions also occasioned by adaptive responses to COVID-19 that reveal lessons for future crisis response. The first is entitled “Digital Distance in times of Physical Distancing: ICT Infrastructure and Use in Long-term Care Facilities.” This contribution focuses on an increasingly important yet often overlooked segment of the population in developed countries, namely older adults, a population at particularly high risk of social isolation and health problems during the pandemic. Using original survey data based on responses from older Swiss adults in the general population as well as residents and managers within the care facilities, the study finds both relatively low uptake of digital technologies combined with a strong desire among the majority of older adult respondents for better ICT resources and support. In particular, the ICT resources in the long-term care facilities proved inadequate for the residents of these facilities by and large, although there were important differences within the resident population. Many of the residents expressed a desire for more training and technical support, pointing to a general inadequacy of ICT skills support within the facilities. As far as usage habits were concerned, younger and male residents tended to use ICT resources more heavily than their female and older counterparts. The study underlines the inadequacy of ICT resources for this population, even within relatively well-funded facilities located in an affluent country, and provides important lessons to better assist this population in the future.

Just as the COVID-19 pandemic made it more critical for digitally disadvantaged groups, such as older adults, to access online resources, it also had an effect on the uptake of virtual reality (VR) digital technology as well. This is the topic of the next study, entitled “The Perceived Impacts of COVID-19 on Users’ Acceptance of Virtual Reality Hardware: A Digital Divide Perspective.” In this article, the analytical eye turns to the factors behind the adoption or non-adoption of a relatively novel technology—virtual reality hardware—during the pandemic. The analysis applies a social-psychological approach to technology uptake— the TAM framework—alongside digital access factors and variables tracking respondents’ experience of the pandemic to characterize the structure of the determinants of VR adoption. The findings suggest that the intense experiences of quarantine and social isolation precipitated by pandemic lockdowns played an important role in stoking interest in VR technology adoption among respondents. Thus, the study shows the importance of adaptive response that prompts respondents to boost their VR uptake to mitigate their concerns for their health and safety. This revealing case study shows how particular large-scale crises can promote or inhibit the uptake of new technologies in tandem with the individual-level factors commonly associated with technology adoption.

Finally, the issue closes with an eye to tools needed for the future: “[De]Politicizing the Pandemic: Visually Communicating Digital Public Sociology.” Drawing together the themes of the issue, this article shed light on one of the most serious challenges to public health heightened by the pandemic, social media echo chambers, and indicates important resources to combat future infodemics, showing how social media has the power to foster self-reinforcing ideological exchanges and self-contained communication networks. Drawing attention to the potential for digital citizen social science, the authors call for user-accessible visual tools to facilitate a bottom-up response to future crises. As the article contends, such digital visualizations would allow users to map out their own engagements vis-à-vis the larger social landscape of opinions. By doing so, visualizations of social media would give members of the general public a vehicle that invigorates a public sociological imagination and a flourishing digital public sociology.

In closing, across these studies, this issue of *American Behavioral Scientist* takes the measure of how communications and media intersected with COVID-19. Light is shed on both the opportunities provided by digital and social media, as well as emergent digital divides, and potential threats to privacy, knolwedge acquisition, and public health that digital media exacerbated. Therefore, not only does digital media presents a double-edged sword of benefit and cost for society as a whole, but benefits and costs are unequally distributed across society along various axes of differentiation. Nevertheless, the issue offers reasons for hope that these new technological tools can be leveraged to enhance societal resilience in the face of large-scale crises and rapid societal change.

